# Influence of tumor-associated factors on the treatment selection between partial nephrectomy and ablation therapy for small renal tumors (Review)

**DOI:** 10.3892/mi.2025.247

**Published:** 2025-06-04

**Authors:** Kensuke Bekku, Shota Inoue, Kasumi Yoshinaga, Tomoaki Yamanoi, Yosuke Mitsui, Tatsushi Kawada, Yusuke Tominaga, Takuya Sadahira, Takehiro Iwata, Satoshi Katayama, Shingo Nishimura, Kohei Edamura, Tomoko Kobayashi, Motoo Araki

**Affiliations:** Department of Urology, Okayama University Graduate School of Medicine, Dentistry and Pharmaceutical Sciences, Okayama 700‑8558, Japan

**Keywords:** small renal mass, partial nephrectomy, ablation therapy, tumor location, endophytic tumor

## Abstract

For small renal tumors, nephron-preserving treatment, including partial nephrectomy or ablation therapy, is recommended. According to major guidelines, ablation therapies are advised for patients who are deemed not suitable to undergo surgery due to an advanced age or the presence of comorbidities. However, compared with surgery, ablation therapy can result in superior safety and functional outcomes. The present review discusses the factors affecting decision-making as regards treatment options for small renal tumors. When determining an appropriate treatment option, tumor locations, as well as the condition and preferences of the patient, are considered. Scoring systems, such as the RENAL Nephrometry Score can assist in guiding treatment decisions. However, surgery may be the preferred approach for tumors near major vessels and collecting systems. For endophytic tumors, partial nephrectomy can be challenging due to the difficulty in visualizing intra-parenchymal tumors during the procedure, whereas ablation therapies may be inferior to partial nephrectomy. Although treatment selection for small renal tumors can be affected by tumor location, partial nephrectomy remains the gold standard for numerous cases.

## 1. Introduction

The increasing use of imaging techniques, such as computed tomography (CT), ultrasound and magnetic resonance imaging, has led to an increase in the incidental detection of small renal tumors. For small-scale cancers, a partial nephrectomy (PN) or ablation therapy (AT) is highly recommended to preserve kidney function. ATs, such as cryoablation (CA) and radiofrequency ablation (RFA), have emerged as valuable treatment options (1), whereas PN remains the gold standard for the treatment of small renal tumors, particularly for clinical T1a (cT1a, #x003C;4 cm) tumors. Based on major guidelines, ATs are recommended for selected patients deemed unfit for surgery (2,3); however, ATs can achieve favorable oncological outcomes comparable to those of PN (4,5). As ATs may provide outcomes superior to those of PN in selected patients (6-8), the selection between PN and AT for small renal tumors remains a topic of debate, and is affected by multiple factors, such as patient health, preoperative renal function and tumor-associated factors, such as tumor complexity. Although ATs are less invasive and suitable for patients who are deemed unfit for surgery, establishing the criteria for ‘poor surgical candidates’ is challenging. The decision becomes particularly complex when considering older adults or otherwise healthy individuals or younger patients with multiple comorbidities. As regards recovery time and complications, AT is generally associated with a shorter hospitalization period, a reduced operative time, lower morbidity rates and a more rapid recovery than that observed following surgical options (6-8). These advantages are particularly relevant for patients who wish to minimize their downtime or have other health concerns. Although surgery remains the gold standard for numerous patients, some may prefer the less invasive nature of ATs, particularly for small renal masses, for which observation may also be a reasonable option. In some cases, the discretion and experience of the clinician, as well as patient preferences may influence treatment selection (9).

Tumor location also plays a critical role in determining appropriate treatment strategies. Although basing treatment decisions on tumor-associated factors can provide a holistic approach (10), their effect on the selection of nephron-sparing treatment remains uncertain. The present review discusses how tumor-associated factors affect the oncological and functional outcomes of cT1 renal cancers.

## 2. Scoring systems and treatment selection

High-complexity tumors, such as hilar or endophytic tumors, are associated with a higher risk of negative oncological outcomes and severe complications when treating small renal masses (11). The RENAL Nephrometry Score is commonly used to assess tumor complexity and to aid in treatment approaches for small renal tumors (9-13). The system consists of five factors, and is simple and practical. A previous study supported the usefulness of scoring systems for predicting the oncological and safety outcomes of ATs and PN (14). Schmit *et al* (14) demonstrated an association between the RENAL score and the outcomes of percutaneous ATs. Patients who experienced treatment failure had significantly higher RENAL scores than those in patients without treatment failure (mean, 7.6 vs. 6.7; P#x003C;0.001) (14). Furthermore, patients who experienced major complications had significantly higher RENAL scores than those in patients without (mean, 8.1 vs. 6.8; P#x003C;0.001) (14).

The Preoperative Aspects and Dimensions Used for an Anatomical (PADUA) score is another system used to assess the tumor complexity. Compared to the RENAL score, the system provides more detailed three-dimensional and anatomical information (15). Takahara *et al* (15) demonstrated that trifecta achievement in patients with PADUA scores ≥10 was significantly lower than that in those with scores #x003C;10 (68 vs. 86%). However, the aforementioned studies investigated the outcomes of different interventions using different scoring systems (14,15). To date, to the best of our knowledge, no scoring system has been developed to compare the outcomes of both PN and ATs. Although treatment decisions based on tumor-associated factors ensure a comprehensive approach, their effect on nephron-sparing treatment selection remains uncertain.

## 3. Novel scoring systems to improve prediction for the outcomes of AT

Several novel scoring systems have been developed or modified to predict the outcomes of each treatment more accurately (16). Gahan *et al* (17) proposed a modified RENAL score based on AT outcomes, primarily involving percutaneous ATs. In this scoring system, tumor size was scaled down compared to the original scale, reflecting the general trend that tumors treated with ATs are typically #x003C;4 cm (17). Their findings indicated that the modified score was significantly associated with initial ablation success and recurrence rates. However, this modified system may limit direct comparison in patients undergoing PN (17). Additionally, a previous meta-analysis assessing existing scoring systems found that although these systems could predict warm ischemic time and complications, they failed to predict a trifecta achievement in PN (9). Thus, the usefulness of these scoring systems for predicting oncological and functional outcomes of both treatments remain unclear. The ABLATE (A: Axial tumor diameter, B: Bowel proximity, L: Location within the kidney, A: Angle, T: touching, E: extra) and simplified ABLATE scores were developed to predict the probability of complications and oncological outcomes following AT (16). These scores consider the association between renal tumors and adjacent abdominal organs, particularly the colon and intestines. A previous study identified tumor depth as an independent predictor of treatment failure following AT. Compared to PN, tumor location within the body is a more critical factor in determining the feasibility of AT (16).

## 4. Components of scoring systems and their significance

It may be challenging for any single scoring system to be applied to all small renal tumors to predict outcomes and guide treatment decisions. An independent evaluation of the scoring system components can help interpret the significance of the findings. Larger tumors tend to have higher malignant potential (9). Although previous studies have applied ATs to larger tumors (>4 cm in diameter; cT1b) (18,19), the use of ATs may be challenging, and supporting evidence remains limited. Even in cT1a tumors, larger tumor size is a predictor of poor prognosis (20,21). ATs are generally considered for tumors #x003C;3-4 cm in diameter. Optimal results are typically achieved for tumors sized #x003C;4 cm, with some guidelines supporting ablation as a primary treatment option for tumors #x003C;3 cm in size (2,3).

Tumor location relative to the polar line is also a component of the scoring systems (11). Owing to their proximity to major vessels and the collection system, hilar tumors require careful consideration to minimize significant complications (11). However, major vessels near tumors can undermine the effectiveness of AT. The heat sink effect can reduce the efficacy of RFA, whereas in CA, the increased temperature due to vessel flow can lead to inadequate ablation (22). Furthermore, hilar tumors often exhibit more aggressive malignant potential (9), thereby affecting oncological control.

Proximity to the collecting system may also affect treatment choice. Among ATs, CA appears to have a lower effect than RFA does on the urinary tract (22). However, Lagerveld *et al* (23) demonstrated that proximity to the collecting system significantly predicted postoperative complications following laparoscopic CA. Another study that included various AT devices identified renal sinus proximity as a risk factor of local recurrence (24). Given these findings, PN may be a preferred approach for tumors near major vessels and the collecting system, although the current evidence remains insufficient to draw definitive conclusions (25).

## 5. Treatment selection for endophytic tumors

Endophytic tumors pose additional challenges due to their location within the normal parenchyma. Theoretically, PN may cause greater damage to normal renal tissue than AT, leading to intraoperative bleeding and post-operative renal impairment. Advanced imaging may facilitate the visualization of endophytic tumors, rendering image-guided percutaneous AT a potentially superior approach (26). Percutaneous CA (PCA) is particularly effective for completely endophytic tumors due to its ability to visualize the ice-ball margins. Although several studies support the feasibility and safety of robot-assisted partial nephrectomy (RAPN) for completely endophytic tumors, available evidence is limited (27).

### RAPN for endophytic tumors

With the widespread use of surgical robots in the field of renal cell carcinoma, indications for PN have expanded to include the management of more complex tumors. Previous studies have shown comparable trifecta achievement in RAPN for endophytic, mesophytic and exophytic tumors (28,29). A previous multi-institutional study with the largest cohort found that RAPN for endophytic tumors resulted in longer warm ischemia times (median, 22 vs. 21 vs. 16 min, respectively; P#x003C;0.001), a greater estimated blood loss (median, 130 vs. 185 vs. 170 ml, respectively; P=0.001) and lower trifecta achievement rates (45.5 vs. 50.9 vs. 68.8%, respectively; P#x003C;0.001), than those for mesophytic and exophytic tumors (28). However, these differences were not significant in clinical practice, and comparable positive surgical margins were observed in those cases with no significant difference (4.5 vs. 3.7 vs. 3.9%, respectively; P=0.9) (28). Of all factors including the endophytic status, multivariate analysis identified tumor size as an independent predictor of trifecta achievement (28). Since the first report in 2014(30), several studies have demonstrated the feasibility and safety of RAPN for completely endophytic renal tumors, and the majority of studies defined endophytic tumors as scores of 3 points for the ‘E’ domain in the RENAL nephrometry score, with few local recurrence and low complication rates (Table I) (25,28-34). Moreover, the post-operative loss of renal function in each study was not significant (29-32). By contrast, the trifecta achievement rate varied from 37.8 to 88.5% (25,28-34). This suggests that RAPN for endophytic tumors is still challenging, and performing it in centers with experienced surgeons may lead to improved results (29).

### Image-guided PCA for endophytic tumors

Conversely, previous studies have demonstrated the efficacy of image-guided PCA for completely or partially endophytic tumors (Table II) (35-38). Murray *et al* (35) evaluated imaging-guided CA in 47 patients with completely endophytic renal cancers. During a median follow-up of 54 months, 6 patients experienced local recurrence, and 5 patients (10%) had seven major treatment-related complications. Jensen *et al* (36) recently reported the safety and feasibility of CT-guided PCA in 56 patients with endophytic renal cancer with a small number of major complications. Considering secondary efficacy in treating imaging-guided AT is critical, and Jensen *et al* (36) revealed that 5 patients received a second PCA; 4 patients were successfully controlled, although 2 of 4 (7%) patients experienced local recurrences within 4 years following PCA and needed to undergo salvage PN (36). As regards functional outcomes, Michimoto *et al* (38) demonstrated a significant difference in the mean estimated glomerular filtration rate (eGFR) level between before and after (at 3 months) the procedure (58.1 and 5.35 ml/min/1.73 m^2^, respectively, P=0.01). In their study, transarterial embolization was performed to visualize intra-parenchymal tumors, which may exhibit significance. By contrast, the sub-analysis according to the pre-operative eGFR level (below and above 60 ml/min/1.73 m^2^) revealed no significance between pre- and post-operative eGFR levels (#x003C;60 group: 44.8±9.4 to 38.8±6.8 ml/min/1.73 m^2^, P=0.07; >60 group: 69.6±7.4 to 66.0±8.7 ml/min/1.73 m^2^, P=0.12) (38). Taken together, these results suggest that ATs can preserve kidney function, even in endophytic tumors (33,36,37).

### Direct comparison between RAPN and PCA for endophytic tumors

Despite the advancements in interventional and surgical technologies, data comparing the outcomes of PN and ATs are limited. Park *et al* (39) compared CT-guided RFA with RAPN for cT1a renal cancer using propensity score matching. In their study, RAPN led to superior recurrence-free survival compared with RFA; however, approximately half of the patients treated with RFA had endophytic tumors compared to 27% of those treated with RAPN. The authors concluded that RFA was a reasonable option for treating endophytic tumors (39). A recent study demonstrated that PCA achieved oncological outcomes and superior safety compared with RAPN for completely endophytic renal tumors (26). Moreover, PCA was superior to RAPN in terms of functional outcomes, where changes in eGFR at 1 year were significantly lower. AS regards oncological outcomes, 2 (3.3%) patients treated with RAPN and 6 (6.5%) patients treated with PCA experienced recurrence, with no significant difference (P=0.428); 3 (3.3%) patients treated with PCA experienced metastases, although none of the patients in the RAPN group had metastases (P=0.139). The trifecta achievement for PCA was comparable (58.8%) to that for RAPN (65.3%, P=0.477) (26). A significant limitation of that study was the non-matched patient group with differences in performance status and comorbidities (26). Additionally, there is no unified definition of trifecta achievement between RAPN and PCA. For RAPN, trifecta is defined as no major complications, negative surgical margins, and no significant (≥25%) reduction in baseline eGFR values. For PCA, trifecta was defined as no major complications, no significant (≥25%), reduction in baseline eGFR, and no residual or enhancing mass at 6 months post-treatment (26). To compare these modalities effectively, standardized criteria for treatment success are required.

## 6. Conclusion

Tumor size and location within the kidney are crucial for determining the feasibility and success of PN and AT, and a multidisciplinary approach that considers tumor characteristics, patient conditions and preferences is necessary. Proximity to the collecting system, renal sinus, or other critical structures affects treatment decisions. For completely endophytic tumors, image-guided AT may provide outcomes comparable to those of PN, although further investigations with matched backgrounds are required. Despite the promising results of AT, surgery remains the gold standard in numerous cases, particularly for larger tumors or when long-term oncological control is a priority. Further studies with matched backgrounds for the two treatment types, such as randomized controlled studies, are desirable.

## Figures and Tables

**Table I:** The outcomes of RAPN for completely endophytic tumors: Overview of reported studies.

Authors, year of publication	No. of patients	Tumor size	Follow-up	Oncological outcomes	Complications	Functional outcomes	Trifecta^a^ achievement (%)	(Refs.)
Autorino *et al*, 2014	65	2.5 (SD 1.1) cm	12.6 (SD 1.1) months	3 (4.6%) patients had PSM; 1 (1.6%) patient had local recurrence	1 (1.6%) patient had grade ≥3 complication	The mean difference in eGFR was-9.7 (SD 17.4)	39(60)	(30)
Komininos *et al*, 2014	45	2.6 (range, 1.3-3.7) cm	48 (range, 20-59) months	5 (11.1%) patients had PSM 1 (2.2%) patient had local recurrence	2 (4.4%) patients had grade ≥3 complication	No significant difference in eGFR change at the last follow-up	17 (37.8)	(31)
Kara *et al*, 2016	87	2.8 (range, 2.1-3.7) cm	15.2 (range, 7-27.2) months	No local recurrence	4 (4.5%) patients had major complications	eGFR preservation rate at the last follow-up was 85.2%	NA	(32)
Harke *et al*, 2018	64	2.4 (range, 1.7-3.0) cm	NA	No PSM	10.9% patients had grade ≥3 complications	eGFR preservation rate at the last follow-up was 72.5%	48(75)	(33)
Raheem *et al*, 2019	52	2.8 (SD 1.3) cm	59 (range, 30-73) months	1 (2.3%) patient had local recurrence	2 (3.8%) patients had grade ≥3 complications	eGFR preservation rate at the last follow-up was 88 (80-100)	NA	(34)
Carvonara *et al*, 2021	147	4.2 (SD 2.5) cm	21.6 (SD 20) months	3 (2.9%) patients had local recurrence 4 (3.3%) patients had metastases	7 (17.5%) patients had grade ≥3 complications	The median difference in eGFR was-10.8 (SD21.5)	44 (45.4)	(28)
Motoyama *et al*, 2022	26	1.9 (range, 0.5-4.5) cm	NA	No PSM	2 (7.7%) patients had grade ≥3 complications	NA	23 (88.5)	(29)
Ito *et al*, 2024	76	2.34 (SD 0.84) cm	12 (range, 1-64) months	Cancer-free rate 97.4%	1 (1.3%) patient had grade ≥3 complication	NA	48 (69.6)	(25)

^a^Trifecta was defined as a warm ischemia time #x003C;25 min, the absence of complications, and a negative surgical margin. RAPN, robot-assisted partial nephrectomy; SD, standard deviation; NA, not available; PSM, positive surgical margins; eGFR, estimated glomerular filtration rate (ml/min/1.73 m^2^).

**Table II:** The outcomes of ablation therapies for endophytic tumors: Overview of reported studies.

Authors, year of publication	Intervention	No. of patients	Tumor size	Follow-up	Oncological outcomes	Complications	Functional outcomes	(Refs.)
Park *et al*, 2010	Laparoscopic RFA	17	2.8 (range 1.7-3.7) cm	32.6 (range, 12-51) months	1 (6.7%) patient experienced local recurrence	No major complications	Pre- and post-operative (day 7) mean creatinine levels were 1.31 and 1.43 (P=0.13)	(39)
Michimoto *et al*, 2016	CT-guided PCA	17 (9 completely endophytic and 8 partial endophytic)	2.65 (range 1.2-3.6) cm	15.4 (SD 5.1) months	1 patient experienced local recurrence at 12 months	No major complications	Pre- and post-operative (3 months) mean eGFR levels were 58.1 and 53.5 (P=0.01)	(38)
Murray *et al*, 2019	CT-guided PCA	47	2.5 (SD 0.5) cm	56 (range, 3-128) months	6 patients had local recurrence, and 5 of 6 were successfully treated with re-PCA	5 patients (10%) had major complications	The mean change in serum creatinine on the day of discharge was 0.2 (SD 0.2) mg/dl	(35)
Jensen *et al*, 2024	CT-guided PCA	56	2.57 (SD 0.87) cm	996 (SD 559) days	4 (7%) experienced local recurrence	3 patients (5.4%) had grade ≥3 complications	NA	(36)

RFA, radiofrequency ablation; CT, computed tomography; PCA, percutaneous cryoablation; SD. standard deviation; NA. not available.

## Data Availability

Not applicable.

## References

[1] Choueiri TK, Schutz FA, Hevelone ND, Nguyen PL, Lipsitz SR, Williams SB, Silverman SG, Hu JC (2011). Thermal ablation vs surgery for localized kidney cancer: A Surveillance, Epidemiology, and End Results (SEER) database analysis. Urology.

[2] Campbell SC, Clark PE, Chang SS, Karam JA, Souter L, Uzzo RG (2021). Renal mass and localized renal cancer: Evaluation, management, and follow-up: AUA guideline: Part I. J Urol.

[3] Ljungberg B, Albiges L, Abu-Ghanem Y, Bedke J, Capitanio U, Dabestani S, Fernández-Pello S, Giles RH, Hofmann F, Hora M (2022). European association of urology guidelines on renal cell carcinoma: The 2022 update. Eur Urol.

[4] Pantelidou M, Challacombe B, McGrath A, Brown M, Ilyas S, Katsanos K, Adam A (2016). Percutaneous radiofrequency ablation versus robotic-assisted partial nephrectomy for the treatment of small renal cell carcinoma. Cardiovasc Intervent Radiol.

[5] Yamanoi T, Bekku K, Yoshinaga K, Maruyama Y, Nagao K, Kawada T, Tominaga Y, Umakoshi N, Sadahira T, Katayama S (2024). Propensity score-matched analysis comparing robot-assisted partial nephrectomy and image-guided percutaneous cryoablation for cT1 renal cell carcinoma. Urol Oncol.

[6] Yanagisawa T, Miki J, Shimizu K, Fukuokaya W, Urabe F, Mori K, Sasaki H, Kimura T, Miki K, Egawa S (2020). Functional and oncological outcome of percutaneous cryoablation versus laparoscopic partial nephrectomy for clinical T1 renal tumors: A propensity score-matched analysis. Urol Oncol.

[7] Cazalas G, Klein C, Piana G, De Kerviler E, Gangi A, Puech P, Nedelcu C, Grange R, Buy X, Jegonday MA (2023). A multicenter comparative matched-pair analysis of percutaneous tumor ablation and robotic-assisted partial nephrectomy of T1b renal cell carcinoma (AblatT1b study-UroCCR 80). Eur Radiol.

[8] Chan VW, Osman FH, Cartledge J, Gregory W, Kimuli M, Vasudev NS, Ralph C, Jagdev S, Bhattarai S, Smith J (2022). Long-term outcomes of image-guided ablation and laparoscopic partial nephrectomy for T1 renal cell carcinoma. Eur Radiol.

[9] Bhindi B, Thompson RH, Mason RJ, Haddad MM, Geske JR, Kurup AN, Hannon JD, Boorjian SA, Leibovich BC, Atwell TD, Schmit GD (2017). Comprehensive assessment of renal tumour complexity in a large percutaneous cryoablation cohort. BJU Int.

[10] Bianchi L, Mineo Bianchi F, Chessa F, Barbaresi U, Casablanca C, Piazza P, Mottaran A, Droghetti M, Roveroni C, Balestrazzi E (2021). Percutaneous tumor ablation versus partial nephrectomy for small renal mass: The impact of histologic variant and tumor size. Minerva Urol Nephrol.

[11] Kutikov A, Uzzo RG (2009). The R.E.N.A.L. nephrometry score: A comprehensive standardized system for quantitating renal tumor size, location and depth. J Urol.

[12] Veccia A, Antonelli A, Uzzo RG, Novara G, Kutikov A, Ficarra V, Simeone C, Mirone V, Hampton LJ, Derweesh I (2020). Predictive value of nephrometry scores in nephron-sparing surgery: A systematic review and meta-analysis. Eur Urol Focus.

[13] Ficarra V, Novara G, Secco S, Macchi V, Porzionato A, De Caro R, Artibani W (2009). Preoperative aspects and dimensions used for an anatomical (PADUA) classification of renal tumours in patients who are candidates for nephron-sparing surgery. Eur Urol.

[14] Schmit GD, Thompson RH, Kurup AN, Weisbrod AJ, Boorjian SA, Carter RE, Geske JR, Callstrom MR, Atwell TD (2013). Usefulness of R.E.N.A.L. nephrometry scoring system for predicting outcomes and complications of percutaneous ablation of 751 renal tumors. J Urol.

[15] Takahara K, Sumitomo M, Fukaya K, Jyoudai T, Nishino M, Hikichi M, Nukaya T, Zennami K, Ichino M, Fukami N (2020). Predictors for trifecta achievement of robot-assisted partial nephrectomy in high-complexity tumors (preoperative aspects and dimensions used for an anatomical score ≥10). Asian J Endosc Surg.

[16] Papa M, Biondetti P, Colombo R, Ierardi AM, Angileri SA, Lucignani G, Boeri L, Montanari E, Cardone G, Scagnelli P, Carrafiello G (2021). sABLATE: A simplified ABLATE score for prediction of complications and outcome in percutaneous thermal ablation of renal lesions. Med Oncol.

[17] Gahan JC, Richter MD, Seideman CA, Trimmer C, Chan D, Weaver M, Olweny EO, Cadeddu JA (2015). The performance of a modified RENAL nephrometry score in predicting renal mass radiofrequency ablation success. Urology.

[18] Caputo PA, Zargar H, Ramirez D, Andrade HS, Akca O, Gao TM, Kaouk JH (2017). Cryoablation versus partial nephrectomy for clinical T1b renal tumors: A matched group comparative analysis. Eur Urol.

[19] Shapiro DD, Wells SA, Best SL, Hedican SP, Ziemlewicz TJ, Lubner MG, Hinshaw JL, Lee FT Jr, Jarrard DF, Richards KA (2020). Comparing outcomes for patients with clinical T1b renal cell carcinoma treated with either percutaneous microwave ablation or surgery. Urology.

[20] Cao C, Kang X, Shang B, Shou J, Shi H, Jiang W, Xie R, Zhang J, Zhang L, Zheng S (2022). A novel nomogram can predict pathological T3a upstaged from clinical T1a in localized renal cell carcinoma. Int Braz J Urol.

[21] Kitagawa Y, Nakashima K, Shima T, Izumi K, Narimoto K, Miwa S, Miyagi T, Maeda Y, Kadono Y, Konaka H (2011). Clinicopathological outcomes of clinical T1a renal cell carcinoma by tumor size. Jpn J Clin Oncol.

[22] Bertolotti L, Bazzocchi MV, Iemma E, Pagnini F, Ziglioli F, Maestroni U, Patera A, Natale MP, Martini C, De Filippo M (2023). Radiofrequency ablation, cryoablation, and microwave ablation for the treatment of small renal masses: Efficacy and complications. Diagnostics (Basel).

[23] Lagerveld BW, Brenninkmeijer M, van der Zee JA, van Haarst EP (2014). Can RENAL and PADUA nephrometry indices predict complications of laparoscopic cryoablation for clinical stage T1 renal tumors?. J Endourol.

[24] Maxwell AWP, Baired GL, Iannuccilli JD, Mayo-Smith WW, Dupuy DE (2017). Renal cell carcinoma: Comparison of RENAL nephrometry and PADUA scores with maximum tumor diameter for prediction of local recurrence after thermal ablation. Radiology.

[25] Ito H, Uemura K, Ikeda M, Jikuya R, Kondo T, Tatenuma T, Kawahara T, Komeya M, Ito Y, Muraoka K (2024). Impacts of complete endophytic renal tumors on surgical, functional, and oncological outcomes of robot-assisted partial nephrectomy. J Endourol.

[26] Pandolfo SD, Beksac AT, Derweesh I, Celia A, Schiavina R, Bianchi L, Costa G, Carbonara U, Loizzo D, Lucarelli G (2023). Percutaneous ablation vs robot-assisted partial nephrectomy for completely endophytic renal masses: A Multicenter trifecta analysis with a minimum 3-year follow-up. J Endourol.

[27] Pandolfo SD, Cerrato C, Wu Z, Franco A, Del Giudice F, Sciarra A, Verze P, Lucarelli G, Imbimbo C, Perdonà S (2023). A systematic review of robot-assisted partial nephrectomy outcomes for advanced indications: Large tumors (cT2-T3), solitary kidney, completely endophytic, hilar, recurrent, and multiple renal tumors. Asian J Urol.

[28] Carbonara U, Simone G, Minervini A, Sundaram CP, Larcher A, Lee J, Checcucci E, Fiori C, Patel D, Meagher M (2021). Outcomes of robot-assisted partial nephrectomy for completely endophytic renal tumors: A multicenter analysis. Eur J Surg Oncol.

[29] Motoyama D, Ito T, Sugiyama T, Otsuka A, Miyake H (2022). Comparison of perioperative outcomes among patients with exophytic, mesophytic, and endophytic renal tumors undergoing robot-assisted partial nephrectomy. Int J Urol.

[30] Autorino R, Khalifeh A, Laydner H, Samarasekera D, Rizkala E, Eyraud R, Stein RJ, Haber GP, Kaouk JH (2014). Robot-assisted partial nephrectomy (RAPN) for completely endophytic renal masses: A single institution experience. BJU Int.

[31] Komninos C, Shin TY, Tuliao P, Kim DK, Han WK, Chung BH, Choi YD, Rha KH (2014). Robotic partial nephrectomy for completely endophytic renal tumors: Complications and functional and oncologic outcomes during a 4-year median period of follow-up. Urology.

[32] Kara O, Maurice MJ, Malkoc E, Ramirez D, Nelson RJ, Caputo PA, Stein RJ, Kaouk JH (2016). Comparison of robot-assisted and open partial nephrectomy for completely endophytic renal tumours: A single centre experience. BJU Int.

[33] Harke NN, Mandel P, Witt JH, Wagner C, Panic A, Boy A, Roosen A, Ubrig B, Schneller A, Schiefelbein F (2018). Are there limits of robotic partial nephrectomy? TRIFECTA outcomes of open and robotic partial nephrectomy for completely endophytic renal tumors. J Surg Oncol.

[34] Abdel Raheem A, Chang KD, Alenzi MJ, Lum TG, Ham WS, Han WK, Chung BH, Choi YD, Rha KH (2019). Robot-assisted partial nephrectomy for totally endophytic renal tumors: Step by step standardized surgical technique and long-term outcomes with a median 59-month follow-up. J Laparoendosc Adv Surg Tech A.

[35] Murray CA, Welch BT, Schmit GD, Schmitz JJ, Weisbrod AJ, Callstrom MR, Welch TL, Thompson RH, Kurup AN, Boorjian SA, Atwell TD (2019). Safety and efficacy of percutaneous image-guided cryoablation of completely endophytic renal masses. Urology.

[36] Jensen CG, Dybdahl M, Valtersson J, Mussmann BR, Duus LA, Junker T, Pietersen PI, Lund L, Welch BT, Graumann O (2024). Percutaneous image-guided cryoablation of endophytic renal cell carcinoma. Cardiovasc Intervent Radiol.

[37] Park BK, Gong IH, Kang MY, Sung HH, Jeon HG, Jeong BC, Jeon SS, Lee HM, Seo SI (2018). RFA versus robotic partial nephrectomy for T1a renal cell carcinoma: A propensity score-matched comparison of mid-term outcome. Eur Radiol.

[38] Michimoto K, Shimizu K, Kameoka Y, Sadaoka S, Miki J, Kishimoto K (2016). Transcatheter arterial embolization with a mixture of absolute ethanol and iodized oil for poorly visualized endophytic renal masses prior to CT-guided percutaneous cryoablation. Cardiovasc Intervent Radiol.

[39] Park SH, Kang SH, Ko YH, Kang SG, Park HS, Moon du G, Lee JG, Kim JJ, Cheon J (2010). Cryoablation for endophytic renal cell carcinoma: Intermediate-term oncologic efficacy and safety. Korean J Urol.

